# Effect of moderate hydrostatic pressure on crystallization of palm kernel stearin-sunflower oil model systems

**DOI:** 10.1016/j.crfs.2024.100700

**Published:** 2024-02-16

**Authors:** Federico Basso, Francesco Ciuffarin, Miriam Chiodetti, Marcello Alinovi, Eleonora Carini, Luisa Barba, Lara Manzocco, Maria Cristina Nicoli, Sonia Calligaris

**Affiliations:** a Department of Agricultural, Food, Environmental and Animal Sciences, University of Udine, Via Sondrio 2/A, 33100, Udine, Italy; b Department of Food and Drug, University of Parma, Parco Area delle Scienze, 47/A, 43124, Parma, Italy; c Institute of Crystallography, National Council of Research, 34100, Trieste, Italy

**Keywords:** Lipid crystallization, Moderate hydrostatic pressurization, Rheological properties, Crystal morphology, Fats thermal properties, Synchrotron x-ray diffraction

## Abstract

Lipid crystallization under moderate hydrostatic pressure treatments (200 MPa, 20 °C, 1–24 h) was studied in palm kernel stearin (PS 100%) and its blends with sunflower oil (PS 80, 90 % w/w). Hyperbarically-crystallized samples exhibited significantly higher firmness, elastic modulus and critical stress values as compared to those of the samples crystallized at atmospheric pressure.

These data indicate that moderate hydrostatic pressure favored the formation of a higher amount of small palm kernel stearin crystals as compared to those formed at atmospheric pressure. Pressurization did not affect fat polymorphism, but was able to enhance nucleation instead of crystal growth.

This work clearly demonstrated the efficacy of moderate hydrostatic pressure in steering lipid crystallization, opening interesting possible applications of high-pressure processing technology in the fat manufacturing sector.

## Highlights

• Palm kernel stearin-sunflower oil (80–100% stearin) systems were crystallized under pressure.
• Pressurization (200 MPa) improved samples firmness and rheological properties.
• Pressurized samples solid fraction was higher and organized as smaller crystals.
• Palm stearin polymorphism was not affected by hydrostatic pressurization.
• Pressurization induced the formation of small, disordered, highly strained crystallites.

## Graphical abstract

**Figure:**
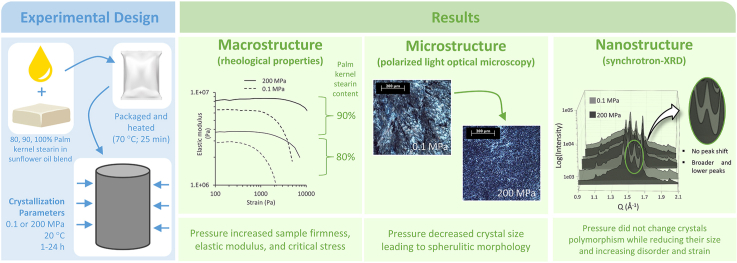

## 1 Introduction

Solid lipids, such as animal fats, tropical oils, margarine, and shortenings, are multipurpose ingredients of many food formulations, playing a specific role in food texture, rheology, and sensory characteristics. The mechanical and rheological properties of fats depend on the structure of the fat crystal network, which enables them to fulfill their intended purposes (Acevedo et al., 2011). At the nanoscale level, triacylglycerols (TAG) crystallize into three main possible polymorphic forms, namely α, β′, and β, having an increasing thermodynamic stability, respectively (Sato and Ueno, 2011). These crystals tend to cluster together, resulting in the formation of larger aggregates that interact, ultimately creating a continuous network. Within this network, the crystal agglomerates are stabilized by van der Waals forces giving structure to the liquid oil entrapped within (Marangoni et al., 2012). Therefore, the investigation of fats structure at different dimensional scales is of great importance to understand the underlying mechanism governing the sensory and technological properties of fat-rich foods. To this regard, it has been widely demonstrated that both composition and processing conditions affect fat crystal network structure (*e.g*., crystal size, morphology, and interactions among crystalline particles) and, thus, its technological functionality (Acevedo and Marangoni, 2015; Ramel et al., 2016). By employing appropriate processing conditions, the crystalline structure of a fat matrix can be effectively controlled and handled. The control of the cooling temperature profiles and the application of shear strain fields are commonly employed as methodologies to control fats crystallization processes (Mishra et al., 2023; Rebry et al., 2021; Sato et al., 2013). More recently, high-intensity ultrasound technology has been proposed to improve the physical and functional properties (*e.g.*, texture, mouthfeel) of lipids (Wagh et al., 2016). Additionally, some studies also highlighted that such objective could be achieved by applying high-pressure processing (HPP) (Zulkurnain et al., 2016b). The capability of this technology to steer fat crystallization depends on the effect of hydrostatic pressure on both the thermodynamics and the kinetics of the phenomenon. From a thermodynamic point of view, pressure favors fats crystallization based on the Le Chatelier Principle. The latter states that the application of pressure to a system in which a physical, chemical or biological phenomenon is occurring will shift the equilibrium of such event towards the state in which the system occupies the least possible volume (Martinez-Monteagudo and Saldaña, 2014). Since the specific volume of lipids in the solid state is lower as compared to the liquid state, pressure will always favor the former (Zulkurnain et al., 2016b). This effect of hydrostatic pressure can be quantified in terms of an hyperbarically-induced *in-situ* increase in fats melting temperature, which is described by the Clausius-Clapeyron equation (Hiramatsu et al., 1992; Brown, 2023; Zulkurnain et al., 2016b). Considering that fats melting is associated with largely positive changes in both molar volume (ΔV = 6–41 mL mol^−1^) and enthalpy (ΔH = 100–300 kJ mol^−1^), the melting temperature of fats can be substantially increased (10–20 K MPa^−1^) when these materials are in the pressurized state (Hagemann and Rothfus, 1983; Hiramatsu et al., 1989; Zulkurnain et al., 2016b). From a kinetic point of view, due to the negative volume change associated to fat solidification, the rate of fat crystallization exponentially increases with pressure according to the Eyring equation (Zulkurnain et al., 2016b). Coherently with these theoretical principles, previous studies have examined the influence of hydrostatic pressure on lipid crystallization, revealing its potential not only in boosting the phenomenon but also in promoting the formation of smaller crystals. In particular, Zulkurnain et al. (2016a, 2019) studied the effect of hydrostatic pressure ranging from 100 to 600 MPa on the crystallization of a blend comprised by soybean oil and different concentrations (10–30%, w/w) of fully hydrogenated soybean oil. The Authors reported that high pressure promoted an increase in crystallization rate, a decrease in crystal size, and the formation of the β polymorph instead of β’ polymorph observed in unpressurized sample. On this aspect and considering the reduced literature available, it should be noted that the effect of high hydrostatic pressure on fat polymorphism seems to depend on the matrix composition. In particular, Kościesza et al. (2010), Tefelski et al. (2008) and Vella et al. (2021) showed that crystallization under pressure greatly favored the α polymorphic form in oleic acid, triolein and milk cream, respectively. Contrarily, Oh and Swanson (2006) observed no significant morphological changes by applying up to 600 MPa during cocoa butter crystallization.

Based on the relevant literature, the feasibility of hydrostatic pressurization to steer fat crystallization is certainly worthy of further investigation in view of its possible scaling up. It should be noted that HPP is already regarded as a mature technology, with a continuously growing number of commercial working units applied as an alternative to thermal pasteurization (Aganovic et al., 2020). Currently, various manufacturers produce HPP equipment operating at pressures even up to 600 MPa. However, their high price has been already pointed out by many Authors as the main limitation of a worldwide commercial application (Cacace et al., 2020). Future innovations are actually needed to increase the economic sustainability of HPP equipment. In this regard, it should be noted that as the pressure required for the process decreases, its costs also decrease, primarily due to the decrease in the vessel wall thickness required to withstand pressure (Bermejo-Prada et al., 2017; Cacace et al., 2020). Based on these considerations, the application of moderate hydrostatic pressure processing (<200 MPa) could represent an efficacious and cost-effective way to steer fat crystallization and techno-functionality. Nevertheless, this topic is still very scarcely explored.

The goal of this research was to study the crystallization behavior of fats under moderate pressure treatments. In this regard, model systems based on palm kernel stearin were selected based on the technological relevance of this matrix in the confectionary industry (Pande et al., 2012). Samples consisted of pure stearin (PS 100%, w/w), and stearin blended with sunflower oil (PS 80 and 90% solid fat, w/w) to take into account the influence of solid fat content. Samples were melted (70 °C), crystallized under moderate hydrostatic pressure (200 MPa, 20 °C), and analyzed for: mechanical (compressive dynamometry), rheological (small amplitude oscillatory rheology) and thermal (differential scanning calorimetry) properties, microstructure (optical microscopy), solid fat content (low resolution ^1^H nuclear magnetic resonance), and crystal structure (synchrotron X-ray diffractometry). Analogous samples crystallized at atmospheric conditions (0.1 MPa, 20 °C) were used as references.

## 2 Materials and methods

### 2.1 Sample preparation

Samples were prepared by mixing palm kernel stearin (kindly donated by a local company specialized in shortenings for bakery purposes; fatty acid composition is reported in Table S1) and sunflower oil (Giglio Oro®, Deoleo, Madrid, Spain), obtaining blends with 80, 90 and 100% (w/w) palm kernel stearin content. Samples were initially set at 70 °C for 25 min in a thermostatic bath. Twenty-gram aliquots of each sample were heat-sealed into polyethylene/ethylene-vinyl-alcohol/polypropylene pouches (5 × 15 cm; 80 μm thickness, water vapor permeability <1 g m^−2^ 24 h^−^ ^1^; Niederwieser Group S.p.A., Campogalliano, Italy) with minimal headspace (Orved, VM-16, Musile di Piave, Italy). Before pressurization, packaged samples (5 × 15 × 1 cm) were heated again at 70 °C for 25 min to ensure complete melting and to delete the memory of crystallization.

### 2.2 Fatty acid composition

Trans-esterification of triglycerides to obtain fatty acid methyl esters (FAME) was performed adapting the method of Sukhija and Palmquist (1988). Briefly, palm kernel stearin was mixed with 2 mL of hexane and 3 mL of methanolic HCl (10% v/v acetyl chloride in anhydrous methanol, Sigma-Aldrich, Milan, Italy) in 20 mL glass tubes. The tubes were sealed with Teflon lined caps, heated at 70 °C for 2 h, and cooled to room temperature. Anhydrous K_2_CO_3_ and Na_2_SO_4_ were added to the mixture to remove moisture, and FAME were extracted with 2 mL hexane (Sigma-Aldrich, Milan, Italy). Samples were incubated for 30 min at room temperature, and centrifuged at 1000×*g* for 10 min at 20 °C. The upper lipid layer was removed, concentrated under N_2_ flow, re-diluted in hexane, and transferred into GC vials for injection. Gas chromatography-mass spectrometry was performed with a 7820A GC system equipped with a 7693A autosampler and automatic split/splitless injector, using a 5977E MSD (Agilent Technologies, Santa Clara, CA, USA) in EI mode (70 eV) as detector. One-microliter aliquots of each sample were injected in split mode (1:100). Separations were achieved on a DB-23 fused silica capillary column (60 m length, 0.25 mm internal diameter coated with 0.25 μm ﬁlm thickness) (Agilent Technologies, Santa Clara, CA, U.S.A.). The GC oven program was as follows: 100 °C for 15 min; 100–200 °C at 5 °C/min; 200 °C for 2 min; 200–205 °C at 0.5 °C/min, 205 °C for 2 min; 205–240 °C at 5 °C/min. After each run, the temperature was set at 245 °C for 2 min for column cleaning. Between runs, an equilibration of 2 min at 100 °C without injected samples was applied. Helium (6.0 purity) at a constant ﬂow rate of 1.0 mL/min was used as carrier gas. The temperatures of transfer line, ion source and quadrupole were set at 230, 240 and 150 °C, respectively. Analyses were performed in full scan mode (m/z 50–600, 3 microscans per second) after a solvent delay of 7 min. FAME were identified by comparing their mass spectra with those from the National Institute of Standards and Technologies Mass Spectral Library. Fatty acids composition was expressed in relation to the relative abundance (peak area %) of each fatty acid methyl ester.

### 2.3 Hyperbaric crystallization

A hydrostatic pressure unit assembled by Comer Srl (Bologna, Italy) was used. It consisted of a screw-capped water-tight steel vessel (maximum pressure: 200 MPa; capacity: 2 L, Hystat, Slaithwaite, Huddersfield, UK) pressurized by a Haskel International high-pressure pump (Burbank, CA, USA). The pressure-mediating fluid was an aqueous solution containing 0.2% (w/w) potassium sorbate and 0.2% (w/w) sodium benzoate (Carlo Erba Reagents Srl, Milan, Italy) to prevent mold proliferation in the fluid reservoir tank. The pressurizing fluid temperature was maintained at 20 ± 1 °C by placing the working unit in a temperature-controlled room. The plastic pouches containing the melted samples (70 °C) were anchored to 10 g-weights and positioned inside the steel vessel. Samples were immediately pressurized (50 MPa min^−1^) at 200 MPa. This pressure was thus reached within 4 min, and maintained for increasing time from 1 to 24 h. After depressurization, all samples were further kept in the vessel for 24 h at 0.1 MPa at 20 ± 1 °C before analysis to allow samples complete setting.

### 2.4 Cooling rate

Control samples crystallized at 0.1 MPa were equipped with a thermocouple data logger (Digital 2 Channel K-Type Thermometer Thermocouples, Gain Express Holdings Ltd., Hong Kong) to monitor samples temperature. For technical reasons, it was not possible to measure temperature during pressurization.

### 2.5 Firmness

Firmness was measured by a uniaxial compression test using an Instron 4301 (Instron LTD., High Wycombe, UK). Samples were removed from pouches without further modification and tested at 20 °C using a 6.2 mm diameter cylindrical probe mounted on a 1000 N compression head at a 25 mm/min crosshead speed. Force-distance curves were obtained from the compression tests and firmness was taken as the maximum force (N) required to penetrate the sample for 2 mm.

### 2.6 Rheological analysis

The rheological properties of samples were determined using a controlled stress rheometer (Haake Rheostress 6000, Thermo Scientific, Karlsruhe, Germany). Aliquots of about 4 g of sample were gently transferred on a 35-mm parallel-plate geometry system, and the measuring gap and temperature were set at 2 mm and 20 °C, respectively. Samples were equilibrated for 5 min before testing to allow internal stress relaxation. Viscoelastic properties were evaluated using oscillatory stress sweep and frequency sweep tests. The former was carried out to determine samples linear viscoelastic region (LVR), that was defined as the stress domain in which samples storage modulus (G′) did not decrease more than 10%. The stress sweep was performed by applying a stress ramp from 1 to 10000 Pa at a frequency of 1 Hz. Samples critical stress (σ*) was identified as the shear stress required to induce a 10% reduction in G′. The frequency sweep test was carried out at a constant stress belonging to the LVR, by decreasing the oscillatory frequency from 10 to 0.1 Hz. Storage modulus (G′), loss modulus (G″), and loss tangent (tan δ = G''/G′) were measured, and their values at 1 Hz were considered for sample comparison.

### 2.7 Differential scanning calorimetry (DSC)

A DSC-3 Star^e^ system (Mettler-Toledo, Greifensee, Switzerland) equipped with Star^e^ software (v.8.10) was used for DSC analysis. Heat flow calibration was achieved using indium (heat of fusion 28.45 J/g). Temperature calibration was carried out using hexane (m.p. −93.5 °C), water (m.p. 0.0 °C), and indium (m.p. 156.6 °C). Approximately 20 mg-aliquots of each sample were weighed in 100 μL aluminum DSC pans and analyzed. An empty pan was used as a reference for all the analyses. The thermal properties of samples crystallized at 0.1 and 200 MPa were determined by heating from 20 to 100 °C at 5 °C/min under nitrogen flow (20 mL/min). Transition peak temperature (T_peak_), onset temperature (T_on_), and melting enthalpy (ΔH) were computed using the STARe software (v. 8.10, Mettler-Toledo).

Additional trials were performed to simulate the cooling profile during the pressurization process. To this aim, the following temperature profile was applied: 70 °C for 25 min, 70–43 °C at −40.0 K min^−1^, 43–32 °C at −6.6 K min^−1^, 32–25 °C at −3.2 K min^−1^, 25–20 °C at −3.0 K min^−1^, 20 °C for 30 min).

### 2.8 Solid fat content (SFC)

^1^H relaxometry was performed at 20.0 ± 0.1 °C using a low resolution ^1^H nuclear magnetic resonance (LR-NMR) spectrometer (20 MHz, the MiniSpec, Bruker Biospin, Milan, Italy). Approximately 4 g of the sample was placed into an NMR tube (10 mm in diameter) and sealed with Parafilm®. ^1^H Free Induction Decay (^1^H FID) experiments were performed to investigate the protons relaxing signals. ^1^H FIDs were acquired using a single 90° pulse, followed by a dwell time of 7 μs, a recycle delay of 3 s, and an acquisition window of 0.1 ms, with 4 scans and 150 data points. The curves were fitted with the bi-Gaussian method (Eq. (1)) proposed by Declerck et al. (2018):

Eq. 1 $${FID}_{fit}={FID}_{Solid}∙e^{-0.5{\left(\frac{t}{T_{2,Solid}^{*}}\right)}^{2}}+{FID}_{Liquid}∙e^{-0.5{\left(\frac{t}{T_{2,Liquid}^{*}}\right)}^{2}}$$

The model fit was done with the *fitting* function, available in Matlab R2022 (Matlab, Mathworks, Natick, U.S.A.). For the fit, different parameters were used. For FID_Solid_ and FID_Liquid_, the maximum and minimum value of the FID signal was used, respectively, restricted to positive values. A value of 13 μs was selected as starting value for T*_2;Solid_. Moreover, a minimum and a maximum value of 5 μs and 22 μs for T*_2;Solid_, respectively, were specified to obtain physically realistic values for the T_2_ value of solids. Finally, a starting value of 2 ms was selected for T*_2;Liquid_.

For the solid fat content (SFC) estimation, Eq. (2) was used:

Eq. 2 $$SFC=\frac{{FID}_{Solid}}{{FID}_{Solid}+{FID}_{Liquid}}∙100$$

### 2.9 Polarized light microscopy (PLM)

Samples were prepared by depositing a small amount of sample onto a glass microscope slide covered with a glass coverslip and applying a small pressure to ensure the sample was thin enough for the measurement. Images were taken at 20 °C at 40 × magnification using a Leica EC3 digital camera, elaborated by the Leica Suite Las EZ software (Leica Microsystems, Heerbrugg, Switzerland), and saved in jpeg format.

### 2.10 Synchrotron X-ray diffractometry (XRD)

X-ray diffraction patterns were recorded at the X-ray diffraction beam-line 5.2 at the Synchrotron Radiation Facility Elettra in Trieste (Italy). The X-ray beam emitted by the wiggler source on the Elettra 2 GeV electron storage ring was monochromatized by a Si(111) double crystal monochromator, focused on the sample, and collimated by a pinhole of the radius of 0.2 mm. Analyses were performed at room temperature. Data were collected at a photon energy of 8.856 keV (1.4 Å), by means of a 2M Pilatus silicon pixel X-ray detector (DECTRIS Ltd., Baden, Switzerland). Fifteen bidimensional patterns were collected for each sample and averaged to reduce signal to noise ratio. Calibration was made by means of a LaB6 standard and data were integrated using the software FIT2D (Hammersley et al., 1996). The indexing and the peak profile parameters refinement of the XRD patterns obtained by the crystalline phases were performed using the programs WinPlotr and Checkcell (Laugier and Bochu, 2000; Roisnel and Rodríguez-Carvajal, 2001). Two peaks belonging to the long axis crystallographic direction, identified as 0 2 0 and 0 6 0, were chosen for every one of the three samples crystallized at ambient pressure and of the three samples crystallized at 200 MPa. Their Pseudo-Voigt parameter has been optimized along with full width at half maximum of intensity (FWHM) and position, and this data allowed estimating crystallite volumes and non-uniform strain along the chosen direction, according to Hosemann ideal-paracrystal theory (Enzo et al., 1988; Hindeleh and Hosemann, 1991).

### 2.11 Data analysis

Data were expressed as the mean ± standard deviation of more measurements. The number of measurements and experimental replicates was adapted for each analysis. In particular: sample cooling profile was measured in single on two replicates; mechanical and thermal properties were measured in duplicate on two replicates; SFC was measured in quadruple on single replicates. Statistical analysis was performed by using R v. 4.2.3 (The R Foundation for Statistical Computing). Analysis of variance (ANOVA) was used to determine statistically significant differences among means (*p* < 0.05). Bartlett's test was used to check the homogeneity of variance (*p* ≥ 0.05) and the Tukey-HSD test was used as post-hoc test (*p* < 0.05).

## 3 Results and discussion

### 3.1 Samples cooling

Preliminary trials were initially conducted to gain insight into the thermal behavior of samples during the cooling phase inside the hyperbaric vessel. Since the measurement of temperature during pressurization was not technically possible, the cooling curves of samples containing 80, 90 and 100% (w/w) palm kernel stearin heated at 70 °C were recorded at ambient pressure. As expected, sample temperature rapidly decreased to 20 °C in approximately 4 min (Figure S1 –red dotted line). Then, a very broad temperature peak extending up to 30 min was observed, which was likely caused by the heat released by samples exothermic crystallization. To identify the occurrence of crystallization during sample cooling, the cooling profile reported in Fig. S1 was simulated in DSC equipment (Figure S1 – grey continuous lines). Exothermic crystallization peaks were detected in all samples after 6–8 min at 20 °C, and persisted for up to 20 min. Palm kernel stearin-sunflower oil systems were maintained in the hyperbaric vessel for at least 1 h (and up to 24 h, as reported in section 2.2) after insertion and pressurization to ensure they reached thermal equilibrium. Even if it cannot be excluded that adiabatic heating and cooling occurred upon samples compression and decompression, these events are part of the process and cannot be avoided.

### 3.2 Structural properties

The effect of the application of hyperbaric conditions during fat crystallization was firstly evaluated considering samples structural properties, with particular reference to firmness and rheological parameters (Table 1 and S2, Fig. S2).

As expected, the firmness of the samples increased with increasing palm kernel stearin concentration (Saghafi et al., 2019). However, samples crystallized under pressure showed significantly higher firmness values as compared to samples crystallized at ambient pressure (Table 1). In this instance, further trials were conducted to establish if maintaining samples under pressure for times longer than 1 h could provide additional improvement to their firmness. However, as reported in Table S2, only negligible changes in sample firmness were obtained by maintaining pressurization at 200 MPa even up to 24 h. In agreement with the literature, this indicates the incapability of hydrostatic pressure to further affect solid crystalline structures after their formation (Zulkurnain et al., 2016b). Based on these results, the attention was thus focused on samples maintained in the hyperbaric vessel for 1 h.

**Table 1:** Firmness and rheological parameters (critical stress, σ*; storage modulus, G′ at 1 Hz; loss tangent, tan δ) of samples containing increasing concentrations of palm kernel stearin (80, 90, 100%, w/w) in sunflower oil and crystallized at 0.1 or 200 MPa.

Palm kernel stearin (%)	Pressure (MPa)	Firmness (N)	Critical stress σ* (Pa)	G' × 10^4^ (Pa)	tan δ (dimensionless)
80	0.1	8.62 ± 1.19 ^e^	587.8 ± 21.0 ^c^	240.4 ± 31.3 ^d^	0.124 ± 0.014 ^a^
	200	18.44 ± 1.06 ^c^	1974.5 ± 35.6 ^b^	303.3 ± 29.2 ^c^	0.079 ± 0.014 ^c^
90	0.1	13.93 ± 1.67 ^d^	2169.0 ± 64.9 ^b^	491.9 ± 36.5 ^b^	0.104 ± 0.006 ^b^
	200	33.99 ± 1.66 ^b^	4344.0 ± 48.7 ^a^	681.3 ± 27.1 ^a^	0.051 ± 0.001 ^d^
100	0.1	30.67 ± 3.37 ^b^	n.d.	n.d.	n.d.
	200	59.29 ± 2.19 ^a^	n.d.	n.d.	n.d.

^a-e:^ different letters in each column indicate statistically different means (*p* < 0.05).

^n.d^. Not determinable.

To better understand the effect of hydrostatic pressure on samples crystallization, rheological analyses were carried out under both non-linear (large strain) and linear (small strain) regimes (Fig. S2). It must be highlighted that rheological analyses were not performed on the 100% (w/w) palm kernel stearin samples since they presented the physical properties of a brittle solid.

Oscillatory stress sweep results at 1 Hz are reported in Fig. S2A. In the linear region, both viscoelastic moduli were not stress-dependent (*plateau*) because the applied stresses produced a proportional strain response (reversible deformation). These data were used to determine the critical stress (σ*), corresponding to the shear stress above which structure breakage occurs (Table 1). Increasing the palm kernel stearin concentration and applying pressure caused an increase in σ*. This indicates that the networks formed with high palm kernel stearin content and crystallized under pressure were more capable to withstand mechanical strain without plastic deformation (Patel et al., 2015).

Concerning frequency sweep curves (Fig. S2B), G′ was always higher than G″ in the tested frequency range, indicating that the elastic component was dominant for all the samples. Rheological parameters at a frequency of 1 Hz were used to compare the samples (Table 1). In agreement with firmness data, the increase of stearin content in the blend caused a significant increase in G'. Even in this case, pressurized samples showed G′ significantly higher than the corresponding unpressurized blends. These results are in agreement with those obtained by Zulkurnain et al. (2016a), who reported a G’ increase in a blend of 30% fully hydrogenated soybean oil and soybean oil crystallized under pressure (600 MPa, 10 min, 25 °C). Samples loss tangent (tan δ) was also calculated using G′ and G'' (*i.e.*, loss modulus) values. This parameter is commonly used to describe the structural order (in terms of molecular interactions) in viscoelastic food systems, with lower tan δ values referred to highly structured materials (Albano et al., 2014). As expected, all samples showed tan δ values lower than 1, indicating the predominance of the solid behavior of the systems over the liquid one (Chai et al., 2020). Both the increase in stearin content in the sample and crystallization pressure caused a significant decrease in tan δ.

### 3.3 Solid fat content and thermal profile

**Fig. 1:**
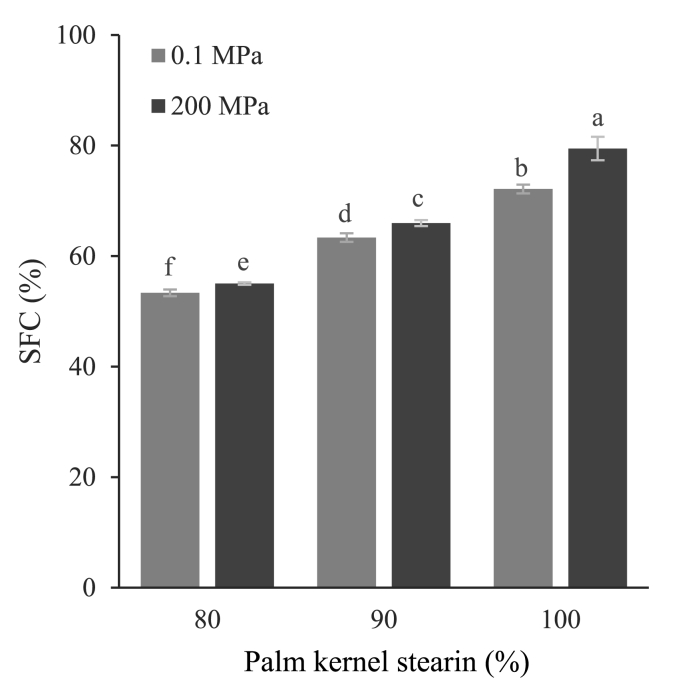
Solid fat content (SFC, %) at 20 °C of samples containing increasing concentrations of palm kernel stearin (80, 90, 100%, w/w) in sunflower oil and crystallized at 0.1 or 200 MPa. a,f: different letters for the samples with equal stearin concentration indicate statistically different means (*p* < 0.05).

To understand the reasons behind the change in mechanical and rheological properties upon pressurization during crystallization, the solid fat content (SFC) at 20 °C of the considered fat blends crystallized at 0.1 and 200 MPa was assessed by LR-NMR (Fig. 1).

**Fig. 2:**
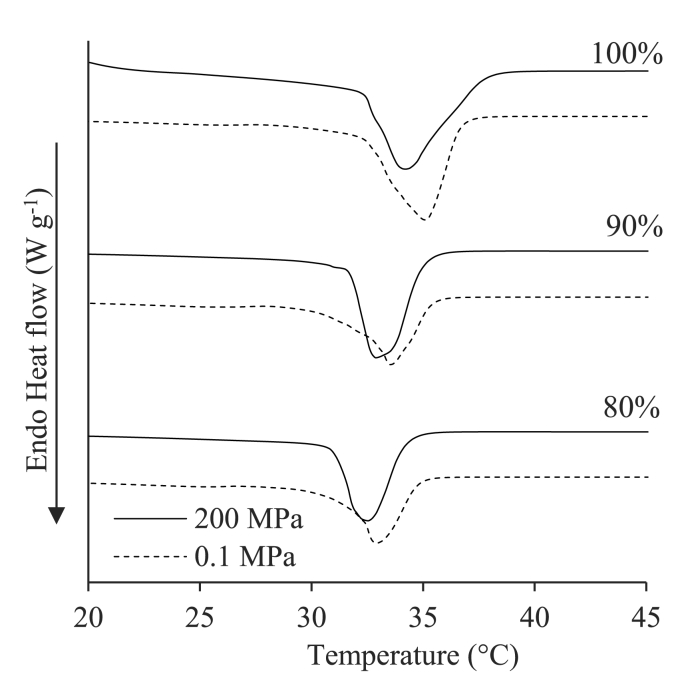
DSC thermograms of samples containing increasing concentrations of palm kernel stearin (80, 90, 100%, w/w) in sunflower oil and crystallized at 0.1 or 200 MPa.

It can be noted that pressurization during crystallization caused a significant increase in SFC (*p* < 0.05). These results strongly indicate that the pressure-induced enhancement of sample mechanical/rheological properties can be associated with an increase of the crystallized palm kernel stearin fraction in the blend. The higher solid fat content upon pressurization was also confirmed by the DSC thermal profile (Fig. 2, Table 2).

**Table 2:** Thermal parameters (T_onset_ and ΔH) of samples containing increasing concentrations of palm kernel stearin (80, 90, 100%, w/w) in sunflower oil and crystallized at 0.1 or 200 MPa.

Palm kernel stearin (%, w/w)	Pressure (MPa)	T_o__nset_ (°C)	ΔH (J g^−1^)
80	0.1	32.2 ± 1.0^a^	73.8 ± 0.1^f^
	200	30.8 ± 0.1^c^	74.9 ± 0.7^e^
90	0.1	32.4 ± 1.4^a^	78.1 ± 0.1^d^
	200	31.2 ± 0.3^b^	85.4 ± 2.0^c^
100	0.1	32.8 ± 0.7^a^	96.7 ± 3.6^b^
	200	32.2 ± 0.5^a^	102.3 ± 1.3^a^

^a-f^ different letters in each column indicate statistically different means (*p* < 0.05).

As expected, all samples showed a single endothermic peak, corresponding to the melting of the crystallized palm kernel stearin fraction. In agreement with the literature, the increase of stearin concentration from 80 to 100% caused a visible peak shift towards higher temperature and an increase in melting enthalpy, due to the higher number of crystallizing components (Zhu et al., 2017). In agreement with Zulkurnain et al. (2019), crystallizing samples under pressure caused a slight shift towards lower temperatures of the melting peak and a concomitant increase in melting enthalpy (ΔH) (Table 2). These results are in agreement with the literature and with SFC data (Fig. 1), clearly indicating the capability of hydrostatic pressure to promote the crystallization of fats while modifying their thermal stability (Zulkurnain et al., 2016b).

### 3.4 Micro and nanostructure

**Fig. 3:**
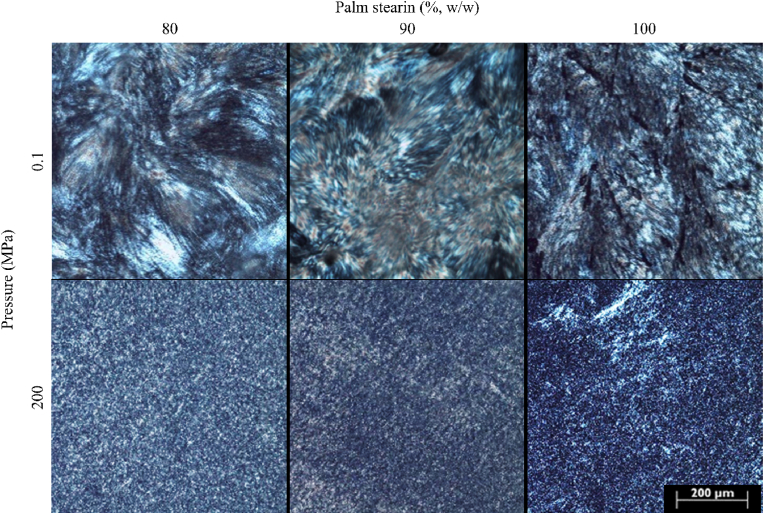
Polarized light microscopy images of samples containing increasing concentrations of palm kernel stearin (80, 90, 100%, w/w) in sunflower oil and crystallized at 0.1 or 200 MPa.

Fig. 3 shows the polarized light micrographs of samples containing 80, 90, and 100% (w/w) palm kernel stearin upon crystallization at 0.1 or 200 MPa.

In agreement with the literature, the sample containing 100% palm kernel stearin and crystallized at 0.1 MPa clearly showed a compact microstructure composed of large needle-shaped crystalline aggregates (Biswas et al., 2017). As visible from Fig. 3, the addition of sunflower oil resulted in larger spacing between crystal aggregates, which was likely due to the displacement of these structures induced by the presence of the liquid phase.

The application of hyperbaric crystallization caused a clear modification of fat crystal network microstructure with the formation of a higher number of evenly dispersed, small crystalline particles. These data, along with those reported in Table 1, Table 2, indicate that pressure favored the formation of a larger amount of smaller, spherulitic crystal aggregates, which formed a more densely packed network as compared to samples crystallized at ambient pressure. This network feature, along with the higher SFC, ultimately led to fat materials with more pronounced solid-like behavior than the control samples. This is in agreement with previous studies highlighting that smaller crystals were associated to the formation of a stronger and more structured network (Campos et al., 2002; Pérez-Martínez et al., 2007).

**Fig. 4:**
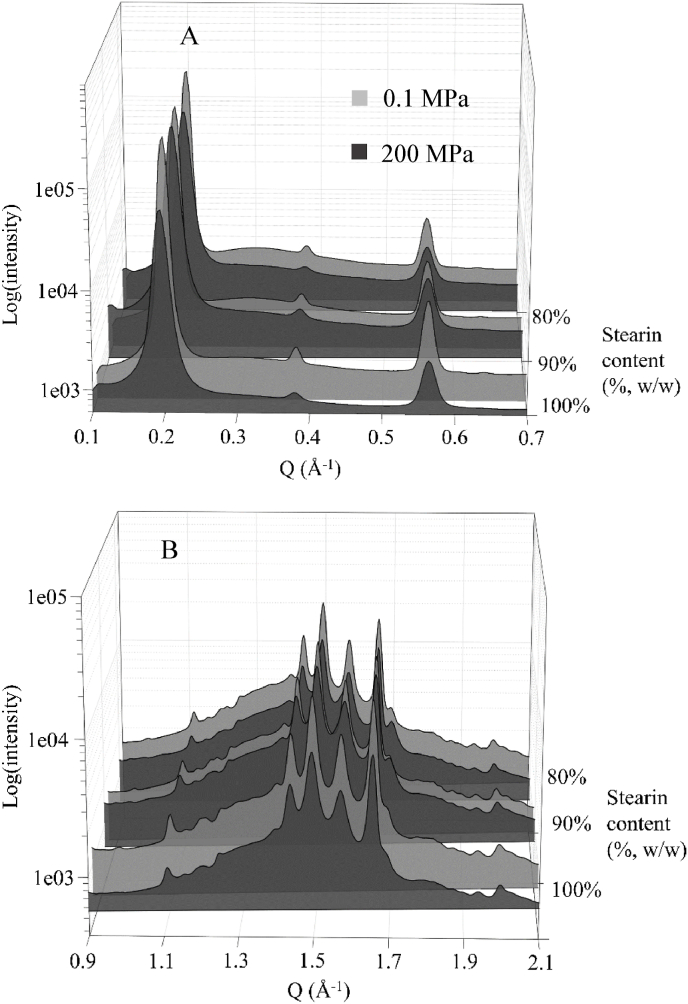
Normalized small-angle (A) and wide-angle (B) diffractograms of samples containing increasing concentrations of palm kernel stearin (80, 90, 100%, w/w) in sunflower oil and crystallized at 0.1 or 200 MPa.

Synchrotron-XRD analysis was performed to better determine the impact of pressurization on crystal morphology. Samples wide- and small-angle XRD diffractograms (WAXD and SAXD), which were produced by the crystal interplanar spacings related to inter-chain (shorter cell dimensions) and chain-length (longer cell dimension and higher reflection orders) distances, respectively, are reported in Fig. 4.

As clearly visible, no peak shifts or appearance of other polymorphs were observed in the WAXD and SAXD region as a consequence of the application of pressure during crystallization. The three small-angle spacings were interpreted as 020, 040 and 060 of a long axis of about 68.5 Å, and the wide-angle set of peaks observed at 3.73, 3.82, 4.03, 4.24, 4.41, 4.53 Å was interpreted as a β′monocline configuration (Van Mechelen et al., 2008).

Despite the same position in the diffractograms, it can be noted that peaks associated to pressurized samples were broader in both WAXD and SAXD regions, as compared to unpressurized samples. This result is typically associated with an increase of the fat crystal network disorder (Ungár, 2004). To quantitatively evaluate the effect of pressure on the structural characteristic of palm kernel stearin crystalline network, diffraction profiles were fitted and elaborated to determine the crystallite size (D_s_), volume-adjusted crystallite size (D_v_), microstrain and paracrystallinity index (Enzo et al., 1988; Hindeleh and Hosemann, 1991) (Table 3).

**Table 3:** Surface-adjusted crystallite size (D_s_), volume-adjusted crystallite size (D_v_), microstrain and paracrystallinity of samples containing increasing concentrations of palm kernel stearin (80, 90, 100%, w/w) in sunflower oil and crystallized at 0.1 or 200 MPa.

Palm kernel stearin (%, w/w)	Pressure (MPa)	D_s_ (Å^3^)	D_v_ (Å) (Å^3^)	Microstrain (−) *10^−4^	Paracrystallinity^a^ (%)
80	0.1	333.1 ± 0.2	364.4 ± 0.4	104 ± 5	3.8 ± 0.2
	200	320.2 ± 0.2	346.1 ± 0.4	104 ± 5	3.6 ± 0.2
90	0.1	393.3 ± 0.3	423.0 ± 0.5	95 ± 5	3.8 ± 0.2
	200	340.9 ± 0.2	368.2 ± 0.4	107 ± 5	4.0 ± 0.2
100	0.1	378.7 ± 0.2	426.6 ± 0.5	86 ± 4	3.4 ± 0.2
	200	306.1 ± 0.2	328.6 ± 0.4	100 ± 5	3.4 ± 0.2

a Lattice fluctuation vector relative to the two successive orders 020 and 060 of the same crystallographic direction.

As visible from the data reported in Table 3, the increase of stearin concentration from 80 to 100% caused an increase in the crystallite size and a decrease in microstrain of crystals formed during cooling under atmospheric conditions. Although counterintuitive at a first glance, this can be associated to crystal growth faster than nucleation phenomena, thus leading to larger crystals with lower interfacial tension (Greiner et al., 2012). When pressure was applied, a decrease of D_s_ and D_v_, with a concomitant microstrain increase, was observed, confirming that the application of pressure caused the formation of smaller crystallites. Interestingly, the effect of pressure resulted much more effective in reducing crystal size as the stearin content increased. It can be inferred that pressurization further favored nucleation instead of crystal growth.

## 4 Conclusions

This study demonstrates the applicability of moderate hydrostatic pressure treatments to steer the crystallization of model systems composed by palm kernel stearin and sunflower oil. Crystallization under pressure (200 MPa, 20 °C, 1 h) significantly enhanced the firmness, the elastic modulus, and the critical stress of all samples. This effect was attributed to the presence of a higher number of small, disordered, highly strained crystals. This behavior was noted in all the considered samples, independently on the content of sunflower oil. It would be interesting in further studies to better investigate the impact of the dilution of the fat phase.

The acquired results indicate the possibility of exploiting hydrostatic pressurization to tailor the techno-functional properties of fat ingredients at the industrial level. Considering that the technology is already widely employed on a large scale within the food sector (*e.g.*, non-thermal pasteurization), its application to fat manufacturing will likely not pose significant technical hurdles. Future studies are needed to demonstrate the applicability of hydrostatic pressure treatments on fat containing foods, such as shortenings, chocolate spreads and baked goods. It is worth noting that the possibility to steer fat properties using hydrostatic pressure could be extended to other industrial context, such as cosmetics and pharmaceuticals. Nevertheless, to the best of our knowledge, these extremely interesting research topics are still completely unexplored.

## Funding

This research did not receive any specific grant from funding agencies in the public, commercial, or not-for-profit sectors.

## CRediT authorship contribution statement

**Federico Basso:** Conceptualization, Investigation, Formal analysis, Data curation, Visualization, Writing – original draft. **Francesco Ciuffarin:** Conceptualization, Investigation, Formal analysis, Data curation, Visualization, Writing – original draft. **Miriam Chiodetti:** Formal analysis, Investigation. **Marcello Alinovi:** Methodology, Validation, Data curation, Writing – review & editing. **Eleonora Carini:** Methodology, Validation, Resources, Data curation, Writing – review & editing. **Luisa Barba:** Methodology, Investigation, Data curation, Visualization, Writing – review & editing. **Lara Manzocco:** Conceptualization, Validation, Resources, Supervision, Writing – review & editing. **Maria Cristina Nicoli:** Conceptualization, Resources, Project administration, Supervision, Funding acquisition, Writing – review & editing. **Sonia Calligaris:** Conceptualization, Validation, Resources, Supervision, Writing – review & editing, Funding acquisition.

## Declaration of competing interest

The authors declare that they have no known competing financial interests or personal relationships that could have appeared to influence the work reported in this paper.

## Data Availability

Data will be made available on request.
