# Supplementary material for: Effect of moderate hydrostatic pressure on crystallization of palm kernel stearin-sunflower oil model systems
Source: Curr Res Food Sci. 2024 Feb 16;8:100700. doi: 10.1016/j.crfs.2024.100700 (PMC10907390; doi:10.1016/j.crfs.2024.100700)
Supplement: Multimedia component 1 [file mmc1.docx]

**TITLE**

**Effect of moderate hydrostatic pressure on crystallization of palm kernel stearin-sunflower oil model systems**

**AUTHORS**

Federico Basso^1^, Francesco Ciuffarin^1^, Miriam Chiodetti^2^, Marcello Alinovi^2^, Eleonora Carini^2^, Luisa Barba^3^, Lara Manzocco^1^*, Maria Cristina Nicoli^1^, Sonia Calligaris^1^

**AFFILIATIONS**

^1^ Department of Agricultural, Food, Environmental and Animal Sciences, University of Udine, Via Sondrio 2/A, 33100 Udine, Italy

^2^ Department of Food and Drug, University of Parma, Parco Area delle Scienze, 47/A, 43124 Parma, Italy

^3^ Institute of Crystallography, National Council of Research, 34100 Trieste, Italy

**AUTHORS E-MAIL**

federico.basso@uniud.it

ciuffarin.francesco@spes.uniud.it

miriam.chiodetti@unipr.it

marcello.alinovi@unipr.it

eleonora.carini@unipr.it

luisa.barba@cnr.it

lara.manzocco@uniud.it*

mariacristina.nicoli@uniud.it

sonia.calligaris@uniud.it

*Corresponding author

**Supplementary material**

Table S1: Fatty acid composition of the palm kernel stearin used in this work.

| Fatty acid | Structure | Content  (peak area %) |
| --- | --- | --- |
| Octanoic acid, methyl ester | C8:0 | 1.63 ± 0.01 |
| Decanoic acid, methyl ester | C10:0 | 2.72 ± 0.04 |
| Undecanoic acid, methyl ester | C11:0 | 0.03 ± 0.00 |
| Dodecanoic acid, methyl ester | C12:0 | 52.52 ± 0.40 |
| Tridecanoic acid, methyl ester | C13:0 | 0.06 ± 0.00 |
| Methyl tetradecanoate | C14:0 | 23.09 ± 0.11 |
| Hexadecanoic acid, methyl ester | C16:0 | 10.05 ± 0.11 |
| Octadecanoic acid, methyl ester | C18:0 | 2.18 ± 0.03 |
| 9-Octadecenoic acid, methyl ester, (E)- | C18:1w9 | 6.74 ± 0.07 |
| 9,12-Octadecadienoic acid, methyl ester | C18:2w6 | 0.90 ± 0.02 |
| Eicosanoic acid, methyl ester | C20:0 | 0.07 ± 0.01 |

Table S2: Firmness of samples containing increasing concentrations of palm kernel stearin (80, 90, 100%, w/w) in sunflower oil maintained in the conditions applied for crystallization (0.1 and 200 MPa, 20 °C, inside the hyperbaric vessel) for increasing time up to 24 h.

| Time  (h) | Firmness (N) | | | | | |
| --- | --- | --- | --- | --- | --- | --- |
|  | 80% (w/w) stearin | | 90% (w/w) stearin | | 100% (w/w) stearin | |
|  | 0.1 MPa | 200 MPa | 0.1 MPa | 200 MPa | 0.1 MPa | 200 MPa |
| 1 | 8.62 ± 1.19^cd^ | 18.44 ± 1.06^b^ | 13.93 ± 1.50^c^ | 33.99 ± 1.66^a^ | 30.67 ± 3.37^c^ | 59.29 ± 2.19^b^ |
| 2 | 7.44 ± 1.31^d^ | 22.17 ± 0.92^a^ | 18.56 ± 2.21^b^ | 34.07 ± 3.72^a^ | 29.98 ± 2.90^c^ | 75.98 ± 6.29^a^ |
| 4 | 7.17 ± 0.59^cd^ | 19.91 ± 1.49^ab^ | 14.41 ± 1.46^c^ | 33.12 ± 0.64^a^ | 30.82 ± 8.39^c^ | 69.02 ± 8.94^ab^ |
| 16 | 8.34 ± 1.24^cd^ | 18.82 ± 3.02^b^ | 13.68 ± 0.49^c^ | 34.90 ± 1.10^a^ | 36.88 ± 2.69^c^ | 67.28 ± 2.36^ab^ |
| 24 | 10.07 ± 0.73^c^ | 18.16 ± 1.74^b^ | 14.43 ± 1.33^c^ | 36.02 ± 2.03^a^ | 32.60 ± 5.70^c^ | 65.63 ± 8.03^ab^ |

^a,d:^ different letters for the samples with equal stearin concentration indicate statistically different means (*p* < 0.05).

Figure S1: DSC thermograms of samples containing increasing concentrations of palm kernel stearin (80, 90, 100%, w/w) in sunflower oil cooled from 70 to 20 °C and further maintained at 20 °C for 25 min. The red dotted line shows the recorded temperature in the hyperbaric vessel.

Figure S2: Changes in elastic (G', circles) and viscous (G'', triangles) moduli as a function of oscillatory stress (A) and frequency (B) of samples containing 80 (empty symbols) and 90% (full symbols) of palm kernel stearin in sunflower oil and crystallized at 0.1 or 200 MPa.
